# Bio-Inspired Optimization Algorithm Associated with Reinforcement Learning for Multi-Objective Operating Planning in Radioactive Environment

**DOI:** 10.3390/biomimetics9070438

**Published:** 2024-07-17

**Authors:** Shihan Kong, Fang Wu, Hao Liu, Wei Zhang, Jinan Sun, Jian Wang, Junzhi Yu

**Affiliations:** 1 The State Key Laboratory for Turbulence and Complex Systems, Department of Advanced Manufacturing and Robotics, College of Engineering, Peking University, Beijing 100871, China; kongshihan@pku.edu.cn; 2 SPIC Nuclear Energy Co., Ltd., Beijing 100029, China; wufang@spic.com.cn; 3 The College of Information Science and Technology, Beijing University of Chemical Technology, Beijing 100029, China; 2023200818@buct.edu.cn (H.L.); 2021210482@buct.edu.cn (W.Z.); 4 National Engineering Research Center for Software Engineering, Peking University, Beijing 100871, China; sjn@pku.edu.cn; 5 The Laboratory of Cognitive and Decision Intelligence for Complex System, Institute of Automation, Chinese Academy of Sciences, Beijing 100190, China; jianwang@ia.ac.cn

**Keywords:** reinforcement learning, improved genetic algorithm, radioactive environment planning, bio-inspired optimization algorithm, combinatorial algorithm

## Abstract

This paper aims to solve the multi-objective operating planning problem in the radioactive environment. First, a more complicated radiation dose model is constructed, considering difficulty levels at each operating point. Based on this model, the multi-objective operating planning problem is converted to a variant traveling salesman problem (VTSP). Second, with respect to this issue, a novel combinatorial algorithm framework, namely hyper-parameter adaptive genetic algorithm (HPAGA), integrating bio-inspired optimization with reinforcement learning, is proposed, which allows for adaptive adjustment of the hyperparameters of GA so as to obtain optimal solutions efficiently. Third, comparative studies demonstrate the superior performance of the proposed HPAGA against classical evolutionary algorithms for various TSP instances. Additionally, a case study in the simulated radioactive environment implies the potential application of HPAGA in the future.

## 1. Introduction

Nuclear energy has been widely applied in various developed countries, as well as in several developing countries, including China [1]. In this situation, a growing number of humans, robots, and other agents are employed to operate the nuclear facilities, which might increase the risk of nuclear exposure [2]. Although nuclear protective equipment can prevent agents from a large amount of radiation dose, it is harmful to human health and robot stability and reliability to work in the radioactive environment [3]. Therefore, with respect to the path planning problem in the radiation environment, one of the crucial goals is to provide an optimal path traversing all the operating points eventually with the lowest cumulative radiation dose [4]. Note that the traversing issue is defined as a multi-objective operating planning problem, which is distinct from the multi-objective optimization problem.

In overhauling or accident response scenarios, people or robots should traverse all the operating points and then return to the origin. Therefore, how to determine an operating sequence with the minimal radiation dose, namely the multi-objective operating planning problem, is important as well for the path planning process. Note that the aforementioned issue is similar to a standard traveling salesman problem (TSP). Wang et al. proposed an improved particle swarm optimization combined with a chaos optimization algorithm to cut the effective radiation dose when agents traverse over all the nodes [5]. Xie et al. combined the improved ACO algorithm and chaos optimization algorithm to solve the multi-objective inspection path-planning problem [6]. Although both methods are demonstrated to be effective in radiation path planning, the multi-objective operating planning problem can be modeled in a more complex way by taking the task difficulty at each operating point, i.e., the operating time, into consideration to be closer to reality. Compared to the classic TSP, the cost between two operating points is not just a simple Euclidean distance but a compound metric including cumulative dose and the consumed operating time. Therefore, the multi-objective operating planning problem can be modeled as a variant of the traveling salesman problem (VTSP).

This paper aims to solve the multi-objective operating planning problem; one primary part is path planning in the radiation environment considering multiple operating points with different operating difficulty levels and multiple radiation sources of different dose rates. Further, a modified genetic algorithm (GA) associated with reinforcement learning (RL), namely the hyper-parameter adaptive genetic algorithm (HPAGA), is provided to solve the radiation VTSP more efficiently. In practical terms, this proposed methodology will prevent people and robots from excessive radiation doses, holding considerable importance, especially as the nuclear power industry construction continues to develop rapidly.

There are three primary contributions listed as follows:

1. A more complicated multi-objective operating planning problem model in the radiation environment is constructed compared to [6]. Specifically, this model considers the operating difficulty level at each operating point ignored entirely in [6], which influences the time to complete each operating task and then the cumulative radiation dose. Therefore, this newly constructed model is closer to the engineering practice.
2. A combinatorial algorithm framework consisting of the bio-inspired optimal algorithm and reinforcement learning is provided, where the hyper-parameters of GA, including crossover probability, mutation probability, and population size, can be adjusted by the RL during the iterative process in order to solve the VTSP more efficiently.
3. Comparative tests between the proposed HPAGA and several classical evolutionary computing algorithms in terms of solving different TSP instances with diverse scales are conducted to demonstrate the superior performance of the proposed hybrid algorithm.

The rest of this paper is organized as follows: Section 2 gives a brief overview of the related work. The model of the multi-objective operating planning problem in the radiation environment is constructed in Section 3. The combinatorial algorithm framework is described in Section 4. A series of comparative experiments between the proposed method and other classical methods are recorded in Section 5. Besides, a case study in a simulated nuclear facilities inspection task is conducted in Section 6. Finally, the conclusion and future work is expounded in Section 7.

## 2. Related Work

Recently, plentiful path planning and operating planning methods have been proposed for radiation environments to minimize cumulative radiation doses during overhauling or accident response stages [7]. Graph searching, as a typical method for path planning, has been employed for radioactive environments. Liu et al. proposed an A* algorithm to plan the walking path with a minimum dose. Similarly, several sampling-based exploration methods have been utilized in the path planning with reducing radiation dose [8]. Chao et al. proposed a grid-based rapidly exploring random tree star (RRT*) method to prevent workers from nuclear exposure as much as possible [9]. Evolutionary computing algorithms and their variants are widely used to solve this issue. For instance, Zhang et al. proposed a hybrid algorithm consisting of an improved ant colony optimization (ACO), A* algorithm, and particle swarm optimization [2,10]. Meanwhile, Lee et al. provided a conflict-based search approach for multi-agents to find respective optimal paths in the radiation environment [11]. The aforementioned methods aim at finding an optimal path from the start point to the destination point neglecting the possible multiple operating points.

Different from the aforementioned planning issues in the radiation environment, this paper focuses on the multi-objective operating panning problem, which is regarded as a VTSP. Note that TSP is a typical combinatorial optimization problem, which belongs to the NP-hard problem [12]. To solve TSP, related algorithms can be roughly classified into three categories, i.e., exact algorithms, heuristic algorithms, and bio-inspired optimization algorithms [13]. Applegate et al. proposed the concord algorithm via modeling TSP as mixed-integer programming problems, where a branch-and-cut algorithm is utilized to solve it [14]. This is one of the best exact solvers to our best knowledge [15]. Meanwhile, LKH-3 is a state-of-art heuristic algorithm for solving TSP, which involves the thinking of local search and k-opt operators to reduce the exploration space [16]. However, both the exact solvers and the heuristic methods are time-consuming to obtain satisfactory solutions. In contrast, bio-inspired optimization algorithms, such as the representative of approximate algorithms, can obtain accepted solutions of TSP with a short running time. There is GA [17,18], wolf search algorithm [19], rat swarm optimizer [20], and so on for solving TSP. Thereinto, GA is a popular optimization technique that mimics the process of natural selection [21]. However, it is difficult to effectively set up the hyper-parameters including crossover probability, mutation probability, the amount of population, and so on [22]. Recently, several hybrid algorithms combined with evolutionary computing algorithms and reinforcement learning have been provided to solve NP-hard problems [23,24]. Inspired by the creative idea of the hybrid algorithm [25], reinforcement learning is employed to adjust the hyper-parameters of GA according to the fitness of the population so as to speed up convergence and avoid the local minimum in this paper.

## 3. Problem Formulation

### 3.1. Radiation Dose Model

**Figure 1:**
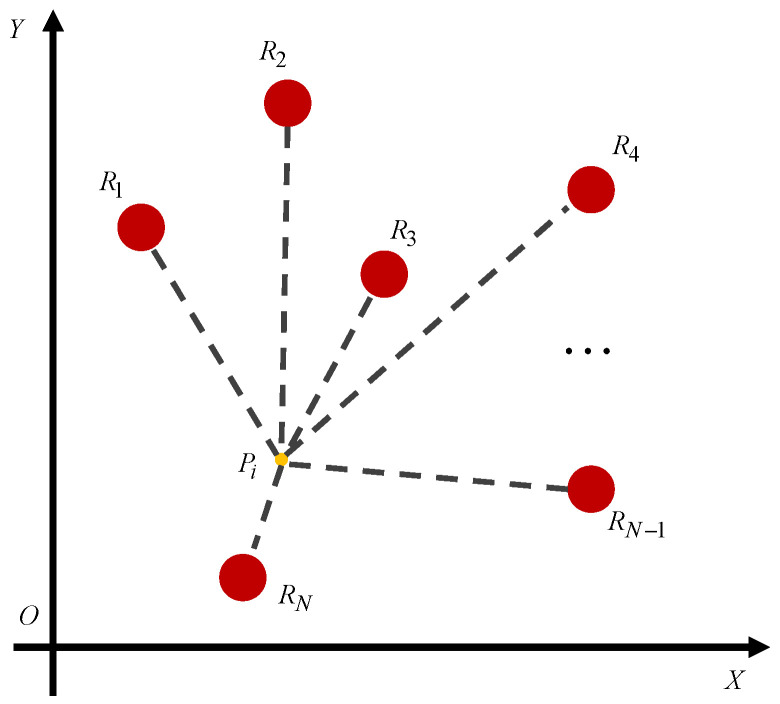
A certain point is infected by the multiple radiation sources.

In the radioactive environment, suppose that there are *N* radiation sources $R_{i}$ with different dose rates, represented by $Dr(R_{i})$, located in the XOY plane as shown in Figure 1. The radiation dose rate derived from each radiation source is inversely proportional to the square of the distance. Therefore, the dose rate of a certain point $P_{i}$ suffering from multiple radiation sources is obtained as

(1) $$D_{r}\left(P_{i}\right)=\sum_{k=1}^{N}\frac{D_{r}\left(R_{k}\right)}{{\left|P_{i}R_{k}\right|}^{2}+1}$$

where $\left|P_{i}R_{k}\right|$ denotes the distance between points $P_{i}$ and $R_{k}$.

**Figure 2:**
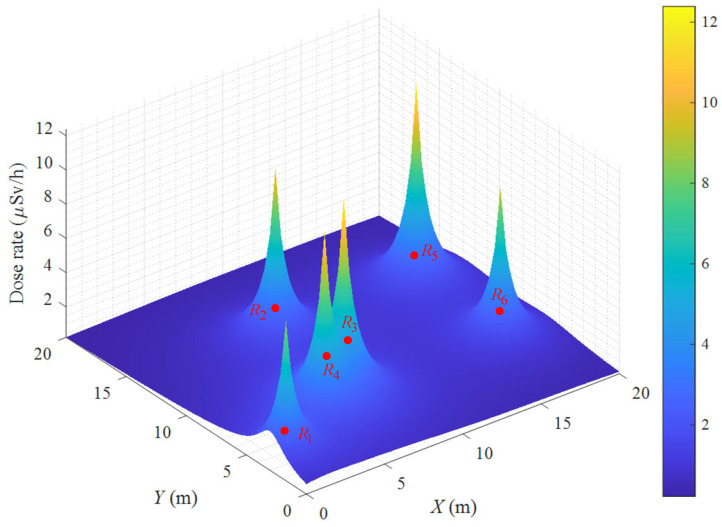
The radiation dose rate map.

The cumulative dose is the crucial reason for causing the harmfulness to people and robots, which is related to the exposure time. With respect to the multi-objective operating planning problem in the radioactive environment, the cumulative dose between two operating points $P_{i}$ and $P_{k}$ consists of primary two parts, namely the locomotion cumulative dose and the operating stay cumulative dose, which is expressed by

(2) $$C_{r}\left(P_{i},P_{k}\right)=C_{rl}\left(P_{i},P_{k}\right)+C_{ro}\left(P_{k}\right)$$

where $C_{rl}\left(P_{i},P_{k}\right)$ means the locomotion cumulative dose between $P_{i}$ and $P_{k}$, and $C_{ro}\left(P_{k}\right)$ denotes the operating stay cumulative dose at $P_{k}$. The radiation dose rate map with six radiation sources is intuitively illustrated in Figure 2.

**Figure 3:**
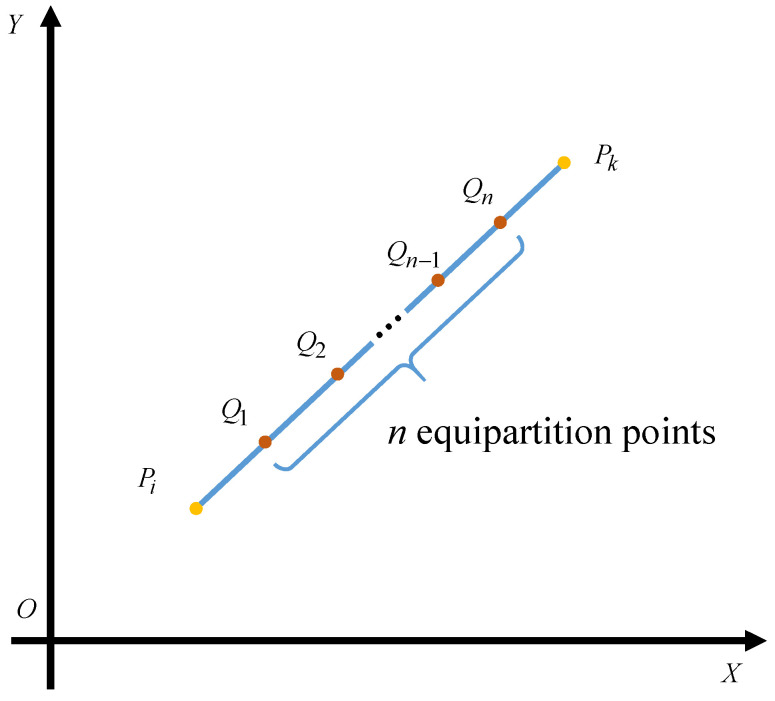
The computing method for cumulative radiation dose between two points.

Concretely, the locomotion cumulative dose is generated during the locomotion from one operating point to the next operating point, which can be calculated by

(3) $$\begin{array}{cc} C_{rl}\left(P_{i},P_{k}\right) & =\sum_{m=1}^{n-1}\frac{\left(D_{r}\left(Q_{m}\right)+D_{r}\left(Q_{m+1}\right)\right)}{2}\times \frac{\left|Q_{m}Q_{m+1}\right|}{v}\\ \; & +\frac{\left(D_{r}\left(P_{i}\right)+D_{r}\left(Q_{1}\right)\right)}{2}\times \frac{\left|P_{i}Q_{1}\right|}{v}+\frac{\left(D_{r}\left(Q_{n}\right)+D_{r}\left(P_{k}\right)\right)}{2}\times \frac{\left|Q_{n}P_{k}\right|}{v} \end{array}$$

where *n* is the resolution factor representing the number of the equipartition points as shown in Figure 3. Besides, *v* denotes the velocity of the agent, which is a constant in this paper.

Meanwhile, the operating stay cumulative dose is derived by

(4) $$C_{ro}\left(P_{k}\right)=D_{r}\left(P_{k}\right)\times T_{s}\left(P_{k}\right)$$

where $T_{s}\left(P_{k}\right)$ represents the cost time during operating at $P_{k}$ which is related to the difficulty of the operating task. Note that the radiation dose model is more complex than [6], for the operating difficulty is taken into consideration when computing the cumulative dose.

### 3.2. VTSP Formulation

In this paper, the multi-objective operating planning problem in the radiation environment is modeled as a variant TSP, where the Euclidean distance between any two nodes is replaced by the cumulative radiation dose. Similar to the typical TSP, the purpose is to find a traversing sequence of operating points with the minimum cumulative radiation dose, where the agent should launch from the origin, pass through every operating point only once, and finally return to the origin.

Suppose that there are *K* operating points $\left\{P_{1},P_{2},\ldots ,P_{K}\right\}$ in the radioactive scenario, the traversing sequence is defined as

(5) $$\Gamma =\left\{B_{o},P_{\left(1\right)},P_{\left(2\right)},\cdots ,P_{\left(K-1\right)},P_{\left(K\right)},B_{o}\right\}$$

where $B_{o}$ means the origin point. Then, the total cumulative radiation dose during the whole process is described as

(6) $$C_{T}\left(\Gamma \right)=\sum_{i=1}^{K+1}C_{r}\left(\Gamma \left(i\right),\Gamma \left(i+1\right)\right)$$

where $C_{t}\left(\Gamma \right)$ denotes the total cumulative dose related to a certain sequence Γ. Furthermore, the optimal sequence with the minimal cumulative dose is obtained by

(7) $$\Gamma^{*}=\underset{\Gamma }{\arg\min}C_{T}\left(\Gamma \right)$$

where exchanging the order of operating points can promote the total cumulative dose to approach the optimal.

So far, the radiation dose model for the multi-objective operating planning problem has been formulated. In the next content, the proposed HPAGA will be introduced to solve this VTSP in an effective way.

## 4. Proposed HPAGA

### 4.1. Algorithm Framework

**Figure 4:**
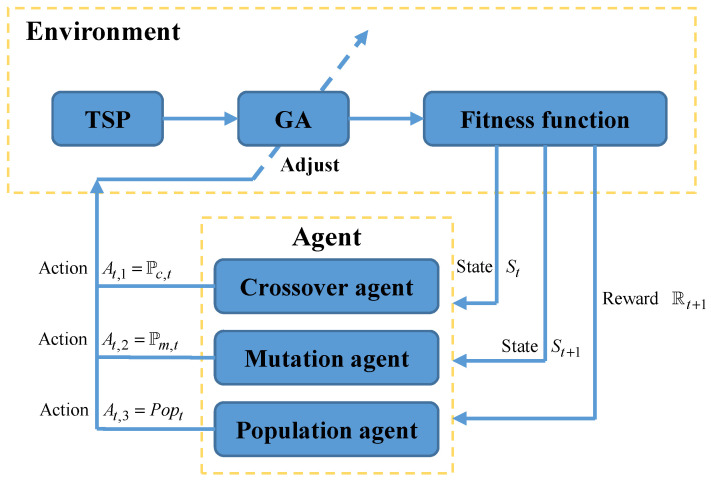
The framework of HPAGA.

HPAGA is a combinatorial optimization algorithm based on the genetic algorithm and reinforcement learning, which can be utilized to solve the TSP and VTSP problems. It mainly consists of two parts, i.e., GA and RL based on Q-learning. Specifically, the hybrid algorithm possesses satisfactory search capability by virtue of the evolution pattern of the genetic algorithm and is able to dynamically adjust the crucial three hyper-parameters of the genetic algorithm including crossover rate, mutation rate, and population size by use of the reinforcement learning. This adaptive mechanism promotes HPAGA to find the optimal path during the search process more quickly and effectively. Note that the proposed algorithm framework is shown in Figure 4. There are three sub-agents in terms of crossover agent, mutation agent, and population agent, which are responsible for adjusting crossover rate $P_{c}$, mutation rate $P_{m}$, and population size Pop of GA, respectively. The reinforcement learning process of HPAGA can be divided into five steps as follows:

- Step 1: The agent obtains the current state $S_{t}$ from GA by calculating the population fitness in a designed way. The regulation of the state space formulation will be expatiated in the following passage.
- Step 2: HPAGA selects and executes the corresponding action $A_{t}=\left[A_{t,1},A_{t,2},A_{t,3}\right]$ according to the action selection policy in reinforcement learning and then adjusts the crossover rate, mutation rate, and population size of the current GA.
- Step 3: Execute the GA with the updated crossover rate, mutation rate, and population size to reach the new state $S_{t+1}$.
- Step 4: Calculate the reward $R_{t+1}$ from state $S_{t}$ to state $S_{t+1}$. The reward estimation method will be introduced in the following passage.
- Step 5: Update knowledge of the agent according to states $S_{t}$, $S_{t+1}$, reward $R_{t+1}$, and action selection policy by Q-learning.

Through a certain number of reinforcement learning iterations, continuously obtaining states, executing actions, receiving reward feedback, and improving policies, HPAGA optimizes the crossover rate, mutation rate, and population size based on past learning experience to elevate the efficiency of GA.

### 4.2. Genetic Algorithm

GA imitates the process of selection, crossover, and mutation in biological evolution, and searches different solutions through continuous evolution to find the individual with the highest fitness.

For each individual of the VTSP problem, it is an operating point sequence as

(8) $$\xi_{i}=\left\{B_{o},P_{\left(1\right)},P_{\left(2\right)},\cdots ,P_{\left(K-1\right)},P_{\left(K\right)},B_{o}\right\},i=1,2,3,...,Pop$$

where $B_{o}$ represents the starting point, $P_{\left(i\right)}$ denotes the operating point, and Pop means the population size.

The initial population is generated randomly through the initialization module, and each individual represents a feasible operating route. The generated routes are accomplished by randomly shuffling the operating point order. This process ensures that the population contains a considerable number of random routes, providing abundant individuals for subsequent optimization processes.

The objective of the VTSP problem is to find the lowest cumulative dose operating sequence for the human or robot. The fitness is determined by calculating the cumulative dose corresponding to each individual. The formula for calculating the fitness $f(\xi_{i})$, i.e., the reciprocal of the summation of the cumulative dose corresponding to each individual, is derived by

(9) $$f\left(\xi_{i}\right)=\frac{1}{C_{T}\left(\xi_{i}\right)}.$$

It is significant to choose an effective crossover operator when solving the VTSP problem. According to the reference [26], the sequential constructive crossover (SCX) operator is utilized to improve the traditional GA. The advantage of the SCX operator is that the generated offspring individuals can relatively retain the high-quality information in the parent individuals, such as superior operating point order and lower cumulative dose, which reduces the possibility of generating unreasonable offspring paths.

### 4.3. Multi-Parameter Adaptive Reinforcement Learning

The reinforcement learning algorithm based on Q-learning is a value-based learning method, which aims to enable agents to learn how to make optimal behavioral decisions in specific environments. The Q-learning algorithm mainly includes several key concepts, i.e., Q-value table, state, action, reward, and policy.

The Q-value table is utilized to record the Q-values learned by the agent, where each row represents a state, each column represents an action, and all values in the initial Q-value table are zero. The Q-value represents the benefit of selecting the corresponding action based on the current state. The Q-value can be calculated based on the current state $S_{t}$, the next state $S_{t+1}$, the selected current action $A_{t}$, the next prospective action $A_{t+1}$, and the next reward $R_{t+1}$, which is expressed as

(10) $$Q\left(S_{t},A_{t}\right)\leftarrow \left(1-\alpha \right)Q\left(S_{t},A_{t}\right)+\alpha \left(R_{t+1}+\gamma \max Q\left(S_{t+1},A_{t+1}\right)\right)$$

where $Q(S_{t},A_{t})$ represents the Q-value of selecting action $A_{t}$ under state $S_{t}$, α represents the learning rate, $R_{t+1}$ represents the reward obtained from state $S_{t}$ to state $S_{t+1}$, γ is the discount factor, and $\max Q(S_{t+1},A_{t+1})$ represents the maximum Q-value in the row of state $S_{t+1}$ in the Q-value table.

With respect to the proposed HPAGA, the state $S_{t}$ of the agent consists of three factors including the relative fitness of the current population’s best individuals $S_{t,1}$, the relative average fitness of the population $S_{t,2}$, and the relative diversity of the population $S_{t,3}$. Therefore, the state for HPAGA is defined as

(11) $$S_{t}=\omega_{1}S_{t,1}+\omega_{2}S_{t,2}+\omega_{3}S_{t,3}$$

where the sub-states are described as

(12) $$S_{t,1}=\frac{\max f(\xi^{p})}{\max f(\xi^{1})},$$

(13) $$S_{t,2}=\frac{\sum_{i=1}^{Pop_{p}}f\left(\xi_{i}^{p}\right)/Pop_{p}}{\sum_{j=1}^{Pop_{1}}f\left(\xi_{j}^{1}\right)/Pop_{1}},$$

(14) $$S_{t,3}=\frac{\sum_{i=1}^{Pop_{p}}\left|f\left(\xi_{i}^{p}\right)-\frac{\sum_{i=1}^{Pop_{p}}f\left(t_{i}^{p}\right)}{Pop_{p}}\right|}{\sum_{j=1}^{Pop_{1}}\left|f\left(\xi_{j}^{1}\right)-\frac{\sum_{j=1}^{Pop_{1}}f\left(\xi_{j}^{1}\right)}{Pop_{1}}\right|}.$$

Note that $\xi_{i}^{1}$ represents the *i* th individual of the initial generation, $\xi_{i}^{p}$ denotes the *i* th individual of *p* th generation, $\xi^{1}$ represents all individuals of the initial generation, $\xi^{p}$ represents all individuals of *p* th generation, $Pop_{p}$ is the population size of *p* th generation, and $Pop_{1}$ represents the population size of the initial generation. Besides, $\omega_{1}$, $\omega_{2}$, and $\omega_{3}$ are positive weights which adjust the importance of three different fitness factors and meet $\omega_{1}+\omega_{2}+\omega_{3}=1$. For example, in the proposed HPAGA, the weights are set to be 0.4, 0.3, and 0.3, respectively.

According to the aforementioned state calculation regulation, the state space will be continuous. In order to ensure a constructible Q-table and a satisfactory convergence speed, the state space is designedly converted to a discrete one. Concretely, the state space is divided into a certain number of intervals. If the value of $S_{t}$ belongs to one interval, $S_{t}$ will be assigned by the characteristic value of this interval. For instance, the state space is divided into 20 intervals. When $S_{t}\in \left[0,0.05\right]$, $S_{t}\leftarrow s\left(1\right)$; when $S_{t}\in \left[0.05,0.1\right]$, $S_{t}\leftarrow s\left(2\right)$; until $S_{t}\in \left[0.95,+\infty \right)$, $S_{t}\leftarrow s\left(20\right)$.

With respect to the action space, the ranges of crossover rate, mutation rate, and population size are divided into a certain number of intervals so as to construct the discrete actions for each agent. The range of crossover rate is from 0.4 to 0.9, the range of mutation rate is from 0.01 to 0.21, and the range of population size is from 50 to 500. Note that the number of intervals can be chosen according to the performance of the algorithm or experiences.

The state transition reward function is designed specifically for each reinforcement learning agent based on the best individual fitness and the population’s average fitness. Therefore, the reward function for the crossover agent is constructed by

(15) $$R_{t+1,\mathrm{cross}}=\frac{\max f\left(\xi^{p}\right)-\max f\left(\xi^{p-1}\right)}{\max f(\xi^{p-1})}.$$

The reward function for the mutation agent is designed by

(16) $$R_{t+1,\mathrm{mutation}}=\frac{\left(\sum_{i=1}^{Pop_{p}}f\left(\xi_{i}^{p}\right)-\sum_{i=1}^{Pop_{p-1}}f\left(\xi_{i}^{p-1}\right)\right)}{\sum_{i=1}^{Pop_{p-1}}f\left(\xi_{i}^{P-1}\right)}.$$

Besides, the reward function for the population agent is a weighted combination of $R_{t+1,\mathrm{cross}}$ and $R_{t+1,\mathrm{mutation}}$ as

(17) $$R_{t+1,\mathrm{population}}=0.5R_{t+1,\mathrm{cross}}+0.5R_{t+1,\mathrm{mutation}}.$$

In this paper, the ϵ-greedy strategy is adopted to select actions. The agent selects the action with the best Q-value via a probability of ϵ based on known information and selects exploration with a probability of 1−ϵ, namely, a random action. The action selection strategy $\pi (S_{t},A_{t})$ is expressed as

(18) $$\pi \left(S_{t},A_{t}\right)=\left\{\begin{array}{cc} \max_{A_{t}}Q\left(S_{t},A_{t}\right), & \mathrm{if}\;\epsilon \leq \epsilon_{o}\\ A_{t}\;\mathrm{randomly}\; & \mathrm{if}\;\epsilon >\epsilon_{o} \end{array}\right.$$

where $\epsilon_{o}\in \left(0,1\right)$ is a threshold value.

## 5. Experimental Results

In this section, experiments on different conventional TSP instances are conducted to verify the superiority of the proposed HPAGA.

### 5.1. Experimental Setup

The test instances in this study are chosen from the widely-used TSP instance library TSPLIB [27]. To demonstrate the effectiveness of our algorithm on datasets of different scales, six instances with different scales, namely att48, berlin52, st70, eil76, gr96, and eil101, are selected. Note that all of them utilize the two-dimensional Euclidean distance metric. With respect to the software and hardware configurations, Python version 3.7.16 is employed for this experiment, and the experimental computer consists of an Intel Core i5-9300H processor, 8 GB of RAM, and Windows 10 operating system.

An overly large population size can result in an unmanageable computational load, while a too-small population may suffer from insufficient diversity. To strike a balance, the initial population size for this task is arbitrarily set at 1000. Too low a crossover rate hinders the proper inheritance of beneficial genes, whereas an excessively high mutation rate can compromise population quality. Consequently, based on empirical observations, the initial crossover rate is set at 0.65 and the initial mutation rate at 0.1 for this task. Drawing from reference [28], the corresponding reinforcement learning parameters are established with a learning rate of 0.75, a discount rate of 0.2, and a greedy rate of 0.85, aiming to foster a synergy between exploration and exploitation for effective and optimized learning.

### 5.2. Ablation Experiment

To verify the effectiveness of the HPAGA in adjusting different hyper-parameters of GA, the ablation experiment is conducted. A comparative study is executed among HPAGA, HPAGA_c (only dynamically adjusting the crossover rate), HPAGA_m (only dynamically adjusting the mutation rate), HPAGA_p (only dynamically adjusting the population size), HPAGA_cm (dynamically adjusting both the crossover and mutation rates), and GA (without applying RL). Each method runs 30 independent epochs with 1000 generations in each epoch on the aforementioned four selected instances. To ensure a fair comparison, the initial population of each dataset was generated with the same random seed so as to produce convincing results.

**Figure 5:**
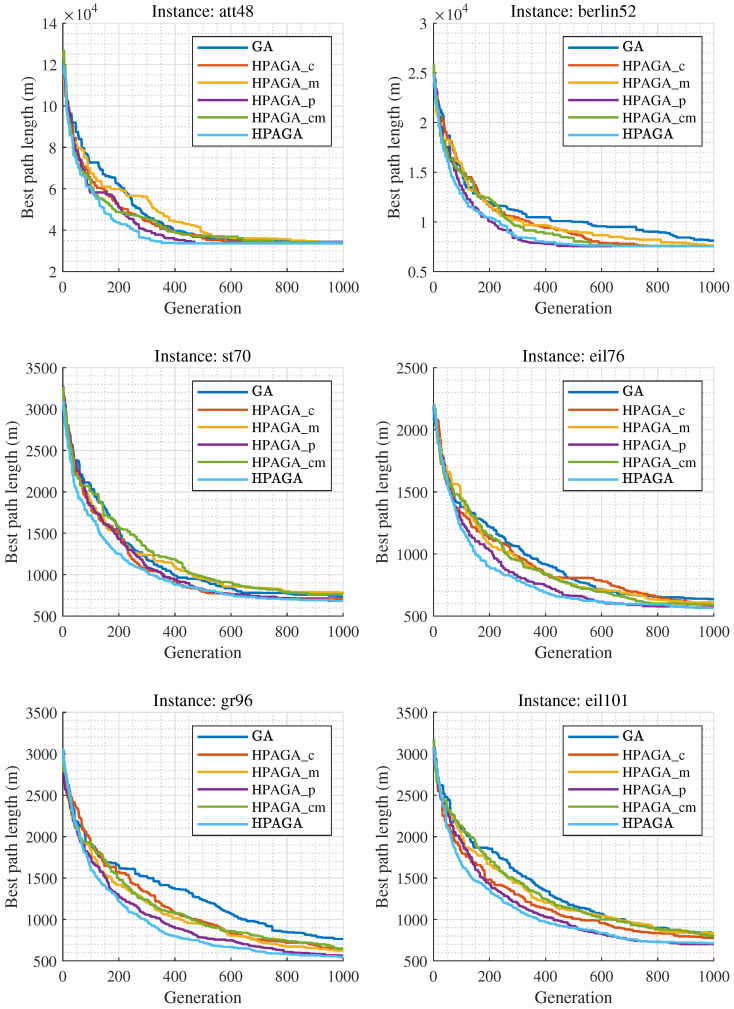
Convergence curves of the six methods in the ablation experiments.

Table 1 shows the results of each method on the four TSP instances. Note that the words **Best**, **Worst**, and **Mean** represent the minimum, maximum, and average cost of the traveling salesman in 30 independent epochs for each algorithm, respectively. **Std** represents the standard deviation of these 30 independent epochs. **Num_c** represents the number of crossover operations, and **Num_m** represents the number of mutation operations for the corresponding algorithm. Figure 5 shows the convergence curves of the best solutions obtained by the six different algorithms on four TSP datasets over 1000 generations in 30 independent epochs. The discussion of the ablation study is expounded from five aspects:

1. Analyzing the comparative results of HPAGA_c and GA, HPAGA_c obtains lower average costs than GA all over the four instances, with fewer crossover operations. This indicates that dynamically adjusting the crossover rate alone can propagate superior genes and improve the overall fitness of the population, then enhancing the performance of GA.
2. Based on the comparative results of HPAGA_m and GA on the four instances, HPAGA_m accomplishes lower minimum costs than GA on att48, berlin52, and eil101 instances, with a fewer number of mutation operations. However, on the st70 instance, HPAGA_m’s minimum and average costs are worse than GA’s. This implies that dynamically adjusting the mutation rate alone can increase population diversity and enhance genetic algorithm performance, but it can also have potentially negative effects due to the influence of mutated individuals in the population.
3. Reviewing the comparative results of HPAGA_p and GA, HPAGA_p acquires lower minimum and average costs than GA in all instances, which demonstrates that the population size agent is effective in improving the classical GA.
4. Examining the results of HPAGA_cm, HPAGA_cm realizes lower minimum and average costs than GA, with fewer crossover and mutation operations. Compared to HPAGA_m, HPAGA_cm reaches a better balance while dynamically adjusting both crossover and mutation rates, promoting population diversity and mitigating the potential negative effects of mutated individuals by propagating superior genes.
5. Among all the comparative algorithms, HPAGA achieves the best performance in most comparative indicators, including the lowest costs and the smallest standard deviation. Note that Figure 5 demonstrates that HPAGA also has the fastest convergence speed.

The ablation study adheres to the principle of variable control. The GA backbones in the experiment have equivalent performance in solving the TSP. Therefore, it is evident that the RL component significantly enhances the TSP-solving performance.

**Figure 6:**
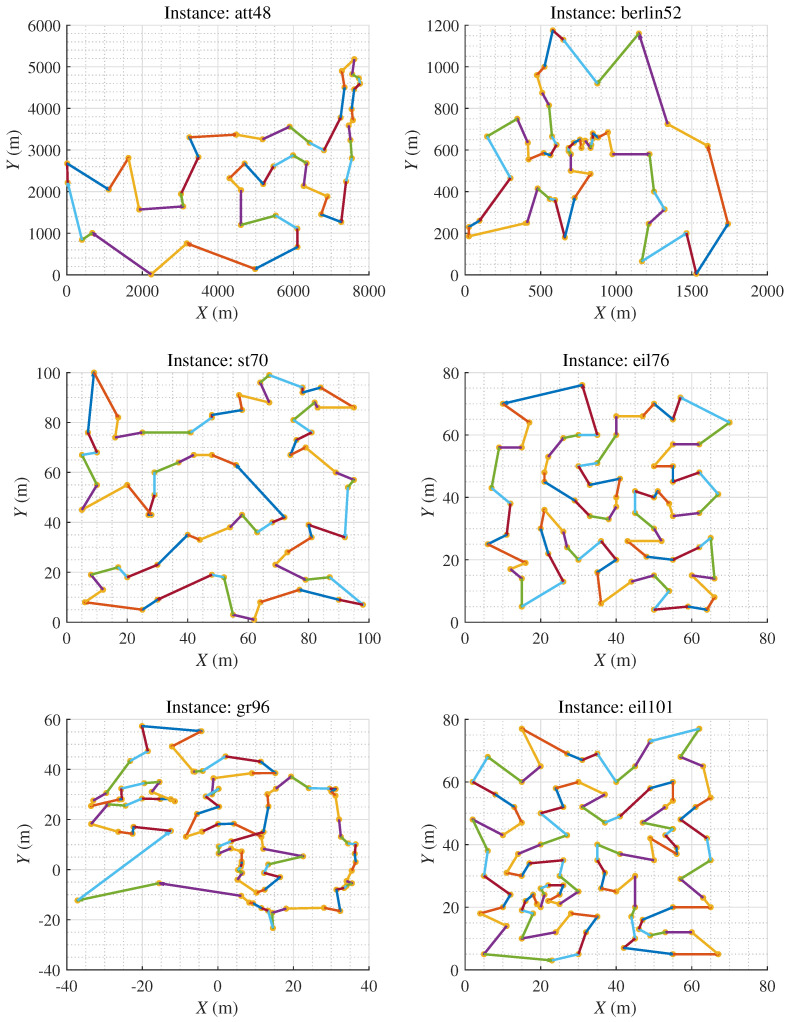
The figure represents the minimum cost path obtained by our HPAGA method in 30 experimental trials.

According to the ablation experiment, it is concluded that in the case of fixed population size, dynamically adjusting the crossover and mutation rates via reinforcement learning strategy assists the hybrid algorithm in obtaining better results than classical GA with fewer genetic operations. In a situation of dynamic adjustments to population size, the appending crossover agent and mutation agent help HPAGA realize comparable or better results than HPAGA_p with fewer genetic operations in the majority of instances. In summary, the comprehensive dynamic adjustment mechanism of HPAGA is the most effective, which significantly improves the performance and stability of GA. As shown in Figure 6, it is demonstrated that in virtue of the proposed HPAGA, the computed path is feasible and basically optimal intuitively.

### 5.3. Comparative Analysis

To verify the performance of the HPAGA algorithm, the comparative analysis of the optimization performance is conducted with several approximate algorithms including ACO, particle swarm optimization (PSO), black hole algorithm (BH), and dragonfly algorithm (DA). The comparative results are listed in Table 2. Note that the computed best solutions of the comparative algorithms source from [29], meanwhile, the configurations of the comparative algorithms are recorded in [30,31].

Based on the comparative results, it can be concluded that the proposed HPAGA algorithm can bridge the remarkable gap between traditional GA algorithms and other evolutionary algorithms. The reason is that HPAGA is an adaptive algorithm involving population fitness, which promotes itself to adjust the parameters to keep on exploring the optimal solutions. However, when the city scale increases, the performance is limited by the number of learning iterations. In the future, more efficient learning tricks will be studied further to improve the capability of solving huge-scale problems.

### 5.4. Limitations

**Figure 7:**
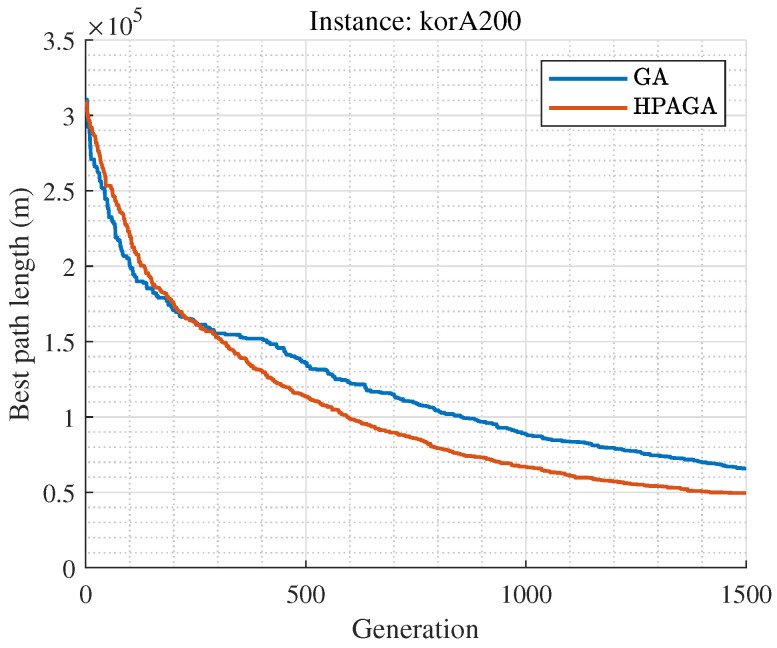
Convergence curves of the GA and HPAGA for korA200.

The HPAGA algorithm proposed in this manuscript performs well in terms of convergence on small-scale Traveling Salesman Problem datasets such as att48, berlin52, st70, eil76, st70, gr96, and eil101, during 1000 iterations of learning. With respect to the large-scale dataset, such as korA200, it is apparent that the performance of HPAGA is superior to the standard GA as shown in Figure 7. However, due to the limitation of the number of iterations, its convergence performance is suboptimal on large-scale datasets. It is shown that the HPAGA algorithm has not yet converged after approximately 1500 iterations on the korA200 dataset, with the fitness still decreasing. In the future, more effective learning techniques will be investigated to improve the capability of solving large-scale problems. Noticeably, the proposed HPAGA might not be the best performer among all the optimization algorithms to our best knowledge, but introduces a novel and valuable hybrid concept to enhance the existing algorithm.

## 6. Case Study in Simulated Radioactive Scenario

**Figure 8:**
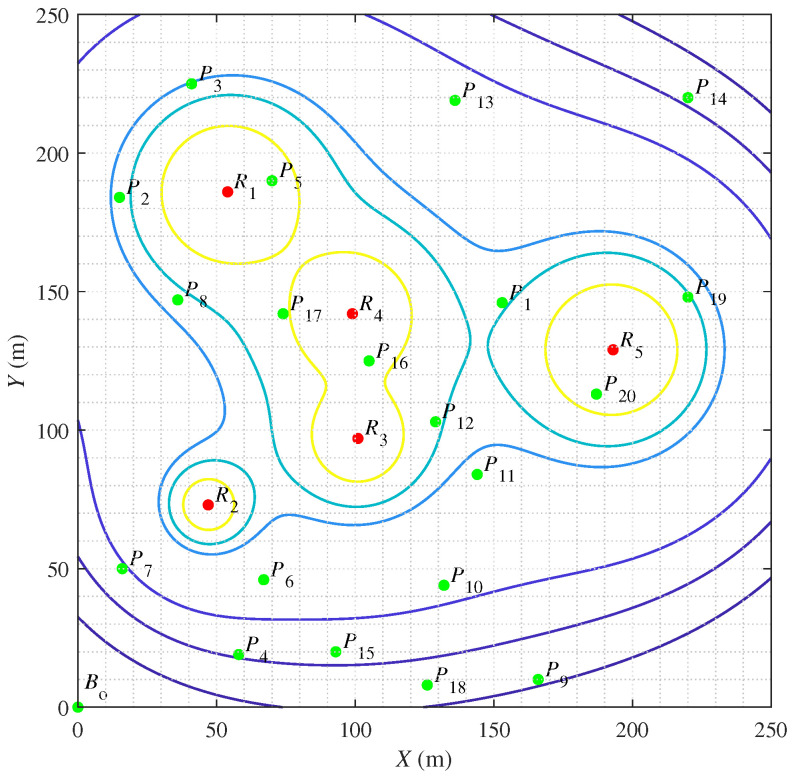
The configuration of the simulated radioactive environment.

In this paper, a case study in the simulated radioactive environment is conducted to demonstrate the feasibility of the proposed HPAGA for the multi-objective operating planning problem. The configuration of the simulated environment is illustrated in Figure 8. Suppose that there are five radiation sources $R_{1}∼R_{5}$ with the radiation dose rate of 1576μSv/h, 240μSv/h, 610μSv/h, 1016μSv/h, and 1550μSv/h, respectively, dispersedly located at the coordinates of (54,186), (47,73), (101,97), (99,142), and (193,129). Note that the contour lines represent the positions with the same value of radiation dose rate. The number of operating points is set as 20. It is different from [6] that the operating difficulty of each operating point is taken into consideration, which is measured by the number of hours consumed at each point. Besides, $B_{o}$ at (0,0) is the starting point. The parameters of these twenty operating points are listed in Table 3. The cumulative dose matrix is defined to describe the cumulative dose between any two points. The value of each element of the cumulative dose matrix in this case is computed according to (3). Apparently, on account of the operating difficulty, the cumulative dose matrix is asymmetric. The case study becomes an asymmetric VTSP.

**Figure 9:**
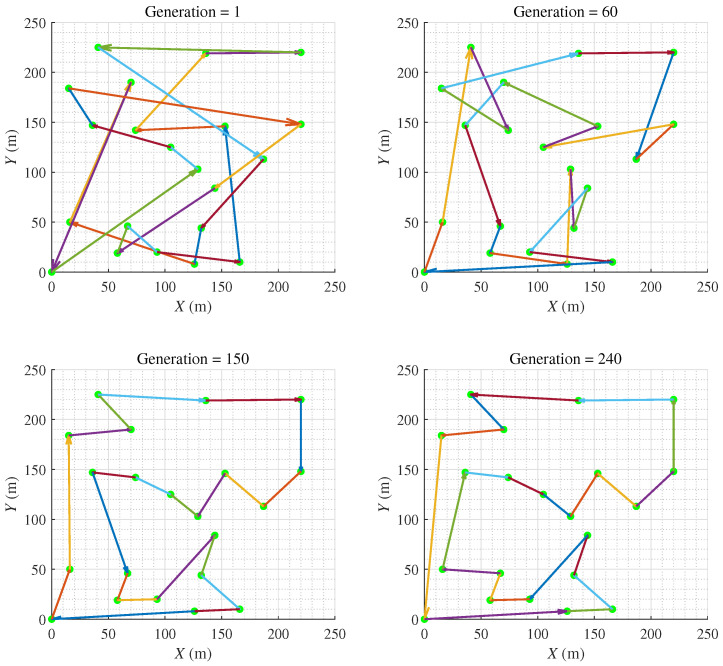
The evolutionary procedure of the HPAGA.

HPAGA is utilized to solve the asymmetric VTSP, the searching procedure for the optimal operating sequence with the increasing generations is exhibited in Figure 9. Note that after the iteration of less than 240 generations, the algorithm has converged to an optimal solution. The results of this simulated case study demonstrate the effectiveness of the proposed HPAGA in solving the multi-objective operating planning problem in the radioactive environment.

## 7. Conclusions and Future Work

This paper introduces a novel multi-objective operation planning model for radioactive environments, accounting for difficulty levels at each operating point to impact operation times and cumulative radiation dose. With respect to the newly designed radiation dose model, a hybrid algorithm framework is proposed that integrates bio-inspired optimization with reinforcement learning, enabling the dynamic adjustment of GA hyper-parameters for efficient VTSP solutions. Noticeably, comparative studies showcase the superior performance of HPAGA against classical evolutionary algorithms for various TSP cases. Furthermore, the case study in the simulated radioactive environment implies the application prospect of HPAGA.

In the future, more efficient learning tricks of the RL part and fresher ideas for hybrid algorithms will be investigated further. Besides, the improved algorithm will be applied to intelligent robots for real-world nuclear scenarios.

## Figures and Tables

**Table 1:** The results of the ablation experiment.

Instance	Index	GA	HPAGA_c	HPAGA_m	HPAGA_p	HPAGA_cm	HPAGA
att48 (33,523) ^1^	Best	34,350	33,900	34,143	33,639	33,929	**33,601**
	Worst	37,462	37,754	39,199	37,256	37,566	**37,136**
	Mean	35,492	35,314	35,740	35,206	35,499	**35,188**
	Std	740	818	1075	766	771	**730**
	Num_c	64,350	58,290	64,350	136,844	58,148	128,976
	Num_m	9876	9889	9604	21,070	9589	18,784
berlin52 (7542) ^1^	Best	8104	7550	7618	7544	7544	**7544**
	Worst	12,398	8791	9371	**8648**	8782	8894
	Mean	10,068	8242	8320	8213	8275	**8116**
	Std	1433	270	320	**252**	289	285
	Num_c	64,351	58,374	64,366	139,140	58,412	130,578
	Num_m	9928	9908	9604	21,406	9586	19,236
st70 (675) ^1^	Best	739	714	764	687	756	**683**
	Worst	927	986	908	766	857	**778**
	Mean	825	803	828	**732**	797	735
	Std	53	51	42	**18**	29	24
	Num_c	64,412	58,250	64,354	139,125	58,444	131,202
	Num_m	9910	9883	9466	21,397	9530	19,287
eil76 (545) ^1^	Best	636	589	611	567	591	**565**
	Worst	930	708	731	**618**	743	620
	Mean	732	652	664	**596**	654	599
	Std	77	25	35	12	34	**12**
	Num_c	64,307	58,341	64,343	137,773	58,614	131,330
	Num_m	9899	9916	9414	21,169	9529	19,151
gr96 (512) ^1^	Best	764	636	619	565	647	**542**
	Worst	1243	890	915	632	792	**644**
	Mean	901	733	760	603	722	**598**
	Std	142	53	66	16	37	24
	Num_c	64,383	58,172	64,333	138,313	58,281	129,260
	Num_m	9909	9911	9372	21,300	9412	18,848
eil101 (629) ^1^	Best	834	778	832	700	796	**713**
	Worst	1343	1064	1178	774	993	**765**
	Mean	1067	873	944	741	865	**736**
	Std	136	57	89	14	43	**14**
	Num_c	64,336	58,108	64,301	138,910	58,330	131,227
	Num_m	9874	9916	9355	21,360	9503	19,322

^1^ The numbers within parentheses below the instance names represent the known optimal distances.

**Table 2:** The comparative results of different methods.

Instance	Method	Best
att48 (33,523) ^1^	GA	34,350
	HPAGA	**33,601**
	ACO	35,231
	PSO	36,996
	BH	34,201
	DA	37,226
berlin52 (7542) ^1^	GA	8104
	HPAGA	**7544**
	ACO	7757
	PSO	9218
	BH	8188
	DA	9401
st70 (675) ^1^	GA	739
	HPAGA	**683**
	ACO	712
	PSO	1031
	BH	723
	DA	797
eil76 (545) ^1^	GA	639
	HPAGA	**565**
	ACO	574
	PSO	804
	BH	566
	DA	625
gr96 (512) ^1^	GA	764
	HPAGA	**542**
	ACO	556
	PSO	1095
	BH	547
	DA	671
eil101 (629) ^1^	GA	834
	HPAGA	**713**
	ACO	725
	PSO	1159
	BH	720
	DA	813

^1^ The numbers within parentheses below the instance represent the known optimal distances.

**Table 3:** The configuration parameters of the operating points.

	$P_{1}$	$P_{2}$	$P_{3}$	$P_{4}$	$P_{5}$	$P_{6}$	$P_{7}$	$P_{8}$	$P_{9}$	$P_{10}$
**Pos. (m)**	(153,146)	(15,184)	(41,225)	(58,19)	(70,190)	(67,46)	(16,50)	(36,147)	(166,10)	(132,44)
**CT (hour)**	0.25	0.3	0.5	0.1	0.15	0.16	0.2	0.2	0.2	0.1
	$P_{11}$	$P_{12}$	$P_{13}$	$P_{14}$	$P_{15}$	$P_{16}$	$P_{17}$	$P_{18}$	$P_{19}$	$P_{20}$
**Pos. (m)**	(144,84)	(129,103)	(136,219)	(220,220)	(93,20)	(105,125)	(74,142)	(126,8)	(220,148)	(187,113)
**CT (hour)**	0.15	0.16	0.2	0.32	0.2	0.25	0.2	0.05	0.2	0.21

Pos. denotes the position of each operating point. CT with the unit of hour represents the consuming time at each point.

## Data Availability

The data generated during the current study are available from the corresponding author upon reasonable request.
